# A Multi-Sensor Data Fusion Approach for Atrial Hypertrophy Disease Diagnosis Based on Characterized Support Vector Hyperspheres

**DOI:** 10.3390/s17092049

**Published:** 2017-09-07

**Authors:** Yungang Zhu, Dayou Liu, Radu Grosu, Xinhua Wang, Hongying Duan, Guodong Wang

**Affiliations:** 1 Key Laboratory of Symbolic Computation and Knowledge Engineering of Ministry of Education, College of Computer Science and Technology, Jilin University, Changchun 130012, China; dyliu@jlu.edu.cn (D.L.); wangxh@ciomp.ac.cn (X.W.); duanhy5513@mails.jlu.edu.cn (H.D.); 2 Department of Computer Engineering, Vienna University of Technology, Vienna 1040, Austria; radu.grosu@tuwien.ac.at (R.G.); guodong.wang@tuwien.ac.at (G.W.); 3 State Key Laboratory of Applied Optics, Chinese Academy of Sciences, Changchun 130012, China

**Keywords:** multi-sensor data fusion, support vector hypersphere, computer-aided diagnosis, trial hypertrophy

## Abstract

Disease diagnosis can be performed based on fusing the data acquired by multiple medical sensors from patients, and it is a crucial task in sensor-based e-healthcare systems. However, it is a challenging problem that there are few effective diagnosis methods based on sensor data fusion for atrial hypertrophy disease. In this article, we propose a novel multi-sensor data fusion method for atrial hypertrophy diagnosis, namely, characterized support vector hyperspheres (CSVH). Instead of constructing a hyperplane, as a traditional support vector machine does, the proposed method generates “hyperspheres” to collect the discriminative medical information, since a hypersphere is more powerful for data description than a hyperplane. In detail, CSVH constructs two characterized hyperspheres for the classes of patient and healthy subject, respectively. The hypersphere for the patient class is developed in a weighted version so as to take the diversity of patient instances into consideration. The hypersphere for the class of healthy people keeps furthest away from the patient class in order to achieve maximum separation from the patient class. A query is labelled by membership functions defined based on the two hyperspheres. If the query is rejected by the two classes, the angle information of the query to outliers and overlapping-region data is investigated to provide the final decision. The experimental results illustrate that the proposed method achieves the highest diagnosis accuracy among the state-of-the-art methods.

## 1. Introduction

Cardiovascular disease is a class of dangerous and troublesome diseases that involve the development of myocardial infarction coronaries, peripheral arterial disease, cerebrovascular disease, cardiomyopathy, heart failure, and endocarditis, etc. It has been the leading cause of death globally [1,2,3]. According to the statistics by the World Health Organization (WHO) [4], 17.5 million people die each year from cardiovascular diseases, an estimated 31% of all deaths worldwide. More than 75% of cardiovascular diseases deaths occur in low-income and middle-income countries. In particular, approximately 3 million Chinese people die from cardiovascular diseases every year, accounting for 40% of all causes of death [5]. Cardiovascular disease deaths resulted in a 4.79 year loss of life expectancy in the Chinese population [5].

The number of patients suffering from cardiovascular disease is increasing dramatically. This leads to a sharp contradiction between the ever-increasing number of patients and limited medical resources. Computer-aided diagnosis (CAD) is a potential solution to alleviate such a contradiction, and it has constantly attracted more and more attention since being proposed [6,7,8,9].

In their technical details, these CAD approaches belong to the sensor data fusion community [10], as the approaches make diagnoses based on fusing the data that is acquired by medical sensors from people. Nowadays, sensor-based e-healthcare systems are attracting increasing attention from both academic and industrial communities. They provides the interpretation of medical images or the reference diagnosis results for clinical staff based on the sensor data from patients. Taking advantage of the considerable development in machine learning algorithms, sensor data fusion has witnessed a widening and deepening of applications in various cardiovascular disease diagnoses [11,12,13]. However, it is a pity that among the numerous diagnosis approaches based on sensor data fusion, those able to effectively address atrial hypertrophy diagnosis are missing. The underlying reason for this is that labelled atrial hypertrophy data are lacking. This causes a serious problem for most learning algorithms because their good performance depends heavily on sufficient training data. For example, supervised learning algorithms based on statistics can derive information concerned with data distribution and density with a large amount of training data.

From the literature, neural networks (NNs) [14,15,16] and support vector machine (SVM) [17,18,19] can solve atrial hypertrophy disease detection. NNs are powerful tools in nonlinear classification and regression, but the formulation of a concrete NN imposes huge and unexpected computational costs in defining the number of layers, the number of neurons in each layer, and the activation functions, etc. Therefore, between these two approaches, SVM is preferred. SVM computes the support vectors based on which a hyperplane is defined to maximize the separation between two classes. Recently, SVM has played an important role in computer-aided diagnosis for cardiovascular diseases including atrial hypertrophy [20,21,22,23,24,25], because it has good generalization performance, a compact structure and a solid theoretical basis. A number of SVM-based works have been proposed in recent years. However, as the practical data to be processed gets confusing, SVM encounters bottlenecks.

The first bottleneck lies in SVM’s hardship in labelling the data within the overlapping margin. SVM is a binary classifier, and it generates the separating hyperplane within the margin between two classes. This means SVM depends completely on the margin between two classes. To the overlapping classes, the margin is covered by data in a mess. It is difficult for SVM to construct a cutting hyperplane that can hold the maximum classification margin. However, it is notable that current medical data may not follow a given distribution, and the difference between patient information and healthy people’s information is getting more and more ambiguous. That is to say, the margin between the patient class and health class is blurred. Consequently, the behavior of SVM is affected by such a data environment. Another bottleneck of SVM is the negligence in handling outliers or novelties. In other words, SVM is not equipped with customized steps to process outliers. In the atrial data, this is a conspicuous issue, because as time goes by the physiological data of patients definitely become ever more complex. Even if two patients suffer from the same disease, there would be a sharp gap between their medical records. It is normal that a patient would exhibit an individual medical record that does not follow the typical characteristics. All these cases correspond to outliers or overlapping-region data, and special steps are required to derive discriminative information from them.

Recently, a diagnosis approach based on sensor data fusion was presented in [25], a locally discriminant SVM (LDSVM) for atrial hypertrophy diagnosis. As an assembling approach, LDSVM consists of SVM and k-Nearest Neighbors (kNN). The former is trained in advance and the latter is started when the confidence of the decision of the SVM is below a threshold. The underlying idea of LDSVM is to append a classifier to modify the unpleasant initial decision. However, there are some problems encoded with LDSVM. Firstly, as [25] indicated, the scenarios when the initial SVM decision fails usually involve datasets with overlapping classes. In such cases, kNN works in the overlapping regions based on a metric derived from the SVM hyperplane function. However, it should be noted that SVM is not good at addressing the overlapping classes, which implies that the hyperplane function produced by SVM would be biased. This leads to a distorted metric on which kNN works, and consequently, poor modification decisions. Secondly, the confidence threshold brings an increase in computational cost, and there is no heuristic for specifying such a parameter. Thirdly, LDSVM ignores the existence of outliers. It is known that medical data are often of high diversity, which means that on the one hand, the margin between the patient group and health group becomes more and more blurred; on the other hand, there exist outliers that are far away from the main occupied regions. If a diagnosis approach ignores the above two aspects, the diagnosis decision will be undesired.

Focused on above issues, in this paper we propose a novel approach for atrial hypertrophy diagnosis, and the approach is named characterized support vector hyperspheres (CSVH). CSVH takes the characters of atrial data (acquired from medical sensors) into consideration and develops two individual hyperspheres for the health class (consisting of the medical records of healthy people) and the patient class (consisting of the medical records of patients), respectively. The hypersphere of the patient class is formulated by a weighted schema, with the aim to identify outliers and overlapping-region data, and consequently, to collect well-defined class information. The hypersphere of the health class keeps furthest away from the patient class hypersphere, with the aim of obtaining the maximum separation between the two groups. When a query arrives, it is labelled according to membership functions defined based on the two hyperspheres. If the query is rejected by the two classes, the angle information between the query and outliers as well as the overlapping-region data is investigated to provide the further decision. To upgrade efficiency, CSVH is equipped with data-adaptive parameterization heuristics.

Instead of constructing a hyperplane as SVM does, CSVH generates hyperspheres to collect the discriminative information, because it is believed that a hypersphere is more powerful for data description than a hyperplane. Moreover, CSVH upgrades the common hypersphere model to the weighted version and the furthest-away version. That assists CSVH in revealing more inner-class information, learning more inter-class difference, and simultaneously allows it to be less affected by outliers and unpleasant data around blurring margins.

The remainder of this paper is organized as follows. Section 2 introduces the experimental data for this study. The idea, procedure and the implementation details of the proposed CSVH are proposed in Section 3. Section 4 discusses the experimental results on benchmark datasets and atrial data. Finally, conclusions and ongoing work are summarized in Section 5.

## 2. Materials

Two real electrocardiogram datasets acquired from medical sensors were used in this study. They are the The Massachusetts General Hospital-Marquette Foundation (MGH/MF) [26,27] and Fantasia [27,28] datasets. There are 250 patient instances in the MGH/MF dataset, and there are 40 healthy people instances in the Fantasia dataset. In both datasets, each instance consists of three files: an annotation file, electrocardio data file and a medical history file. Among them, the second file contains electrocardio curves, which were to be investigated. To obtain a fair comparison, we employed the sampling method in [25] to derive vectors from the electrocardio curves. That is, we chose five heartbeats from the electrocardio curve of one instance to represent the cardiac information. Each heartbeat was described by a fifty-dimension vector. The entries of such a fifty-dimension vector are fifty voltages sampled from the corresponding heartbeat curve. Of course, these fifty voltages were rescaled for normalization.

**Figure 1:**
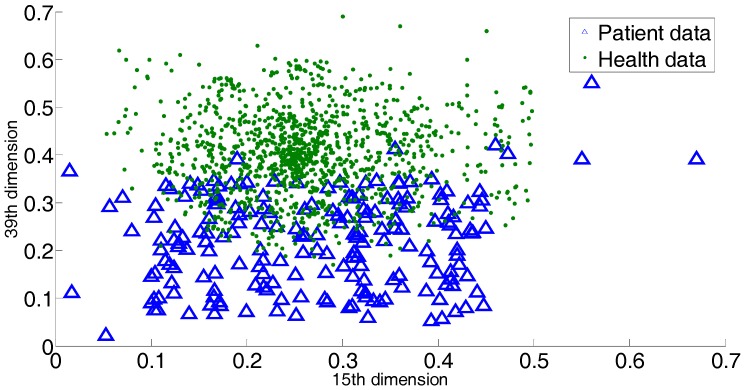
2 dimensions of all the dimensions to briefly show the outliers or overlaps in the atrial hypertrophy datasets.

For atrial hypertrophy, there exists a number of patient records that do not exhibit classical symptoms, i.e., that do not have the classical medical indices of atrial hypertrophy. It is also quite possible that a healthy person shows similar atrial hypertrophy symptoms. This creates great difficulty in providing an accurate diagnosis. It is known that these non-classical data serve as outliers or overlapping data. Thus outliers and overlapping data exist, though the exact number of these is hard to obtain. In Figure 1, we provide 2 dimensions (15th dimension and 39th dimension) of all the dimensions to briefly show the outliers or overlapping samples. The blue triangles indicate the patient sample, and the green dots indicate the sample of healthy people.

## 3. Methods

### 3.1. The Outline of the Proposed CSVH Approach

The CSVH approach consists of two phases: the training phase and the labeling phase.

In the training phase, two characterized hyperspheres for two classes are constructed. Motivated by the observation that the distribution of patient class data for atrial hypertrophy is diverse, the hypersphere of the patient class is equipped with a data-adaptive weighted schema, with the aim to strengthen the presence of outliers and overlapping-region data. Simultaneously, based on the fact that the health group data are relatively denser than the patient data, the hypersphere of the health group keeps furthest away from the patient class, with the aim of obtaining the maximum separation between the two groups.

In the labeling phase, two membership functions are defined based on the two hyperspheres, and they provide the degree of belonging query to the two classes. When a query arrives, its belonging degree to the two classes is first computed. If the two degrees are close enough, the angle information between the query and outliers as well as the overlapping-region data are investigated to refine the membership decision.

The CSVH steps are outlined as follows.

**Training phase:**

(1) Preprocess training of the atrial sensor data.
(2) Construct the weighted hypersphere for the patient class, and obtain *a*, *R*^2^, and *OsetP*.
(3) Construct the furthest hypersphere for the health class, and obtain *b*, *Z*^2^, and *OsetH*.

where *a* is the hypersphere center of the patient class, and *b* is the hypersphere center of the health class; *R* and *Z* are the two hypersphere radii; *OsetH* is the set including outliers and the overlapping-region data of the health class, and *OsetP* is the set including outliers in the patient class.

**Labeling phase:**

(4) For query *Q*, compute its hypersphere-wise membership degrees to two hyperspheres, *G_p_*(*Q*) and *G_h_*(*Q*).
(5) If | *G_h_*(*Q*) − *G_p_*(*Q*)| > *ε_Q_*, label *Q* as the class with higher membership.
(6) If | *G_h_*(*Q*) − *G_p_*(*Q*)| ≤ *ε_Q_*, label *Q* based on the information of the outlier and overlapping-region data.

where *G_p_*(*Q*) and *G_h_*(*Q*) are two membership functions; *ε_Q_* is the threshold to determine whether *Q* is rejected by the two classes.

### 3.2. Characterized Hypersphere of the Patient Class

The diversity in a distribution of patient data requires CSVH to construct a weighted hypersphere to identify the unpleasant data (including outliers and overlapping-region data). For this reason, the formulation of the hypersphere and inner-class data are equipped with large penalty coefficients. This ensures that the hypersphere can cover this data and can develop a well-defined data description of the patient class. Other unpleasant data are equipped with small penalty coefficients, to highlight their presence.

The weighted hypersphere of the patient class is modeled based on the containing hypersphere [9,29] and constructed through optimizing the following objective:

(1) $$\min\;R^{2}+C_{i}\Sigma_{i=1}^{N_{p}}\xi_{i}$$

$$s.t.\;||\varphi (x_{i})-a|{|}^{2}\leq R^{2}+\xi_{i},\;\xi_{i}\geq 0$$

where *N_p_* is the size of the patient class; *ϕ* is the nonlinear map from the input space to the feature space; *ξ_i_* is a slack variable; *a* is the hypersphere center; *R* is the hypersphere radius; and *C* is a penalty coefficient of the slack variable. Introducing a Gaussian kernel *k*(*x_i_*, *x_j_*), the final objective is thus:

(2) $$\min\Sigma_{i,j=1}^{N_{p}}\beta_{i}\beta_{j}k(x_{i},x_{j})-\Sigma_{i=1}^{N_{p}}\beta_{i}k(x_{i},x_{i})$$

$$s.t.\;0\leq \beta_{i}\leq C_{i},\;\Sigma_{i=1}^{N_{p}}\beta_{i}=1$$

where *β_i_* is a Lagrange multiplier.

The hypersphere center *a* is computed as:

(3) $$a=\Sigma_{i=1}^{N_{p}}\beta_{i}\varphi (x_{i})$$

The hypersphere radius is computed by adopting a support vector in the following distance formula:

(4) $$\begin{array}{ll} R{\left(x\right)}^{2} & =||\varphi (x)-a|{|}^{2}\\ & =k(x,x)-2\Sigma_{i=1}^{N_{p}}\beta_{i}k(x,x_{i})+\Sigma_{i,j=1}^{N_{p}}\beta_{i}\beta_{j}k(x_{i},x_{j}) \end{array}$$

The data with *β_i_* = *C_i_* and *ξ_i_* > 0 are outliers and overlapping-region data. They constitute the set *OsetP*.

Then, consider the parameterization of the penalty coefficient *C_i_*. For outliers, they are of large distance to their nearest neighbors. Based on that fact, we consider the distance between *x* and its nearest neighbor, and let that distance serve as the penalty factor:

(5) $$C1_{i}=\exp(-||\varphi (x_{i})-\varphi (x_{inear})|{|}^{2})$$

where *x_inear_* is the nearest neighbor of *x_i_*. The further *x_inear_* is from *x_i_*, the more probable *x_i_* is to be an outlier, and a relatively small penalty coefficient is required. Notice that the distance is computed in the feature space. Introduce a Gaussian kernel, and we have:

(6) $$C1_{i}=\exp(2k(x_{i},x_{inear})-2)$$

For overlapping-region data, some of the neighbors would come from the other class. Therefore, we investigate the ratio of neighbors of the own class to those of the other class. Such a ratio works as another penalty factor, defined as:

(7) $$C2_{i}=\exp(-\frac{\left|OTHER_{i}\right|}{\left|OWN_{i}\right|})$$

where *OTHER_i_* and *OWN_i_* are the sets of neighbors of *x_i_* belonging to the other class and own class of *x_i_*, respectively. |∙| computes the cardinality of the set. The higher the ratio of |*OTHER*| to |*OWN*|, the more probable that *x_i_* is in the overlapping-region data, and consequently, a relatively small penalty factor is required.

Combine the above two factors to form the penalty coefficient customized to *x_i_* as:

(8) $$C_{i}=\frac{1}{2}(C1_{i}+C2_{i})$$

### 3.3. Characterized Hypersphere of the Class of Healthy Subjects

The hypersphere of the health class is characterized by the maximum separation from the hypersphere of the patient class. To keep the furthest distance between the two hyperspheres, the distance between the two hypersphere centers is investigated. Inspired by [30], CSVH constructs the hypersphere of the health class that keeps furthest away from the origin of the feature space, and then maps the hypersphere center of the patient class to the origin to realize the maximum separation between the two hyperspheres.

#### 3.3.1. Rough Model of the Furthest Hypersphere

To keep the hypersphere furthest away from the origin of the feature space, an appendix term is added to the original objective:

(9) $$\min\;Z^{2}-\eta ||b|{|}^{2}+C\Sigma_{i=1}^{N_{h}}\xi_{i}$$

(1) $$s.t.\;||\varphi (x_{i})-b|{|}^{2}\leq Z^{2}+\xi_{i},\;\xi_{i}\geq 0$$

The meanings of *ϕ*, *ξ_i_* and *C* are the same as above. *b* and *Z* are the center and radius of the furthest hypersphere, respectively. *N_h_* is the size of the health class. The parameter *η* determines the importance of ||*b*||^2^ (the distance from *b* to the origin of the feature space) to the model formulation. Denote *γ_i_* as a Lagrange multiplier, and then the final objective is:

(10) $$\min\;\frac{1}{1-\eta }\Sigma_{i,j=1}^{N_{h}}\gamma_{i}\gamma_{j}k(x_{i},x_{j})-\Sigma_{i=1}^{N_{h}}\gamma_{i}k(x_{i},x_{i})$$

$$s.t.\;0\leq \gamma_{i}\leq C,\;\Sigma_{i=1}^{N_{h}}\gamma_{i}=1$$

#### 3.3.2. Refined Model of the Furthest Hypersphere

We mapped the hypersphere center of patient class, *a*, to the origin of the feature space. If *a* is viewed as the origin, then the new axes can be formed. The problem then turns to constructing the common hypersphere for the health class in the space spanned by the new axes. In that new space, there exists a new nonlinear map that is used in the formulation of the hypersphere. The nonlinear map under the new axes is denoted as *ϕ’*. We considered modifying the original Kernel function *k* to a new version *k*’:

(11) $$\begin{array}{ll} k\prime (x,y) & =\varphi \prime (x)\cdot \varphi \prime (y)\\ & =(\varphi (x)-a)\cdot (\varphi (y)-a)\\ & =\varphi (x)\cdot \varphi (y)-a\cdot \varphi (x)-a\cdot \varphi (y)+||a|{|}^{2}\\ & =k(x,y)-2a\cdot \varphi (x)+||a|{|}^{2} \end{array}$$

where *ϕ*’ is the nonlinear map. As mentioned before, *a* = ∑*β_i_φ*(*x_i_*), (*i* = 1 … *N_p_*). Therefore, *k*’ is given by:

(12) $$k\prime (x,y)=k(x,y)-\Sigma_{i=1}^{N_{p}}\beta_{i}k(x_{i},x)-\Sigma_{i=1}^{N_{p}}\beta_{i}k(x_{i},y)+\Sigma_{i,j=1}^{N_{p}}\beta_{i}\beta_{j}k(x_{i},x_{j})$$

Replace *k* with *k*’ in (10), and the furthest hypersphere is obtained.

#### 3.3.3. Parameterization of Balance Coefficient

*η* is the balance coefficient to tradeoff “*Z*^2^” and “−||*b*||^2^” in Formula (9). These two terms essentially determine the volume and separation of the hypersphere for the health class. In this paper, it is derived from the approximate distribution information of the health class in the feature space:

(13) $$\eta =\frac{1}{1+s}$$

where $s=mean\{\sqrt{2-2k\prime (x_{i},x_{j})}|i,j=1\ldots N_{h}\}$.

The motivation for the above parameterization is as follows. *s* computes the average distance among health class members in the feature space, basically indicating the compactness of the health class. If *s* is high, it implies that the members of health class are scattered within a large area. In that case, the term “*Z*^2^” should be strengthened to ensure the tight volume of the health class hypersphere and consequently foster the separation between the two hyperspheres. Thus, a small *η* is required. Alternatively, if *s* is low, it implies that the distribution of the health class is relatively dense. In other words, in that scenario, the separation should be emphasized rather than the hypersphere volume. That is, term “−||*b*||^2^” should be highlighted, and a large *η* is required.

### 3.4. Labeling Phase

With the two hyperspheres in hand, we labelled the query according to its position with respect to two the hyperspheres. Here, CSVH takes the presence of outliers and overlapping-region data into consideration, and develops the membership functions in the below versions:

(14) $$G_{p}(Q)=\exp(-\frac{||\varphi (Q)-a|{|}^{2}}{R^{2}+P^{2}})$$

(15) $$G_{h}(Q)=\exp(-\frac{||\varphi (Q)-b|{|}^{2}}{Z^{2}+H^{2}})$$

where $P^{2}=mean\{||\varphi (x_{u})-a|{|}^{2}|x_{u}\in OsetP\}$, $H^{2}=mean\{||\varphi (x_{v})-b|{|}^{2}|x_{v}\in OsetH\}$.

If |*G_h_*(*Q*) − *G_p_*(*Q*)| > *ε_Q_*, *Q* is labelled as the class member with higher membership values. The threshold *ε_Q_* is specified adaptively as some percentage of the larger membership values:

(16) $$\varepsilon_{Q}=\max\{G_{p}(Q),G_{h}(Q)\}\cdot 10\%$$

If it is typical that |*G_h_*(*Q*) − *G_p_*(*Q*)| ≤ *ε_Q_*, that fact corresponds to two cases. The one is that the query is far away from two classes, which implies that the query is relatively near to the outliers. In this case, labelling the query according to the information of the outliers is reasonable. The second case is that query is located within the margin between the two classes. That means the query is close to the overlapping-region data. In that case, the local information of the overlapping-region data is of discrimination ability to label the query. Therefore, CSVH searches for the most analogous one from *OsetP* and *OsetH* that is of the smallest angle with *Q*. Denote the most analogous one as *QA*, which is defined as:

(17) $$QA=\max_{x}\{<\varphi (Q)\cdot \varphi (x)>|x\in OsetP\cup OsetH\}$$

Then, *Q* is labelled as the same class as *QA*. Therein, the inner product <*φ*(*Q*)·*φ*(*x*)> computes the *Cosine* values of the angle between *Q* and *x* in the feature space. With the different kernel functions in the patient and health classes, *QA* is computed by:

(18) $$QA=\max_{x}\left\{\begin{array}{l} k(Q,x)\begin{array}{cc} & \left(ifx\in OsetP\right) \end{array}\\ k\prime (Q,x)\begin{array}{cc} & \left(ifx\in OsetH\right) \end{array} \end{array}\right\}$$

## 4. Results and Discussion

In this section, we describe the design of the experiments to test the performance of the proposed method, then we present and analyze the experimental results. The experiment environment was Windows 10 with MATLAB R2011b. The kernel function involved in the support vector approaches used the Gaussian kernel. The parameters involved in the experiments, such as the scale of the Gaussian kernel function and penalty coefficients were set by 10-fold cross-validation. Before applying CSVH to atrial hypertrophy data, we ran it on benchmark data to verify its validation and performance.

### 4.1. Experiments on Synthetic Datasets

**Figure 2:**
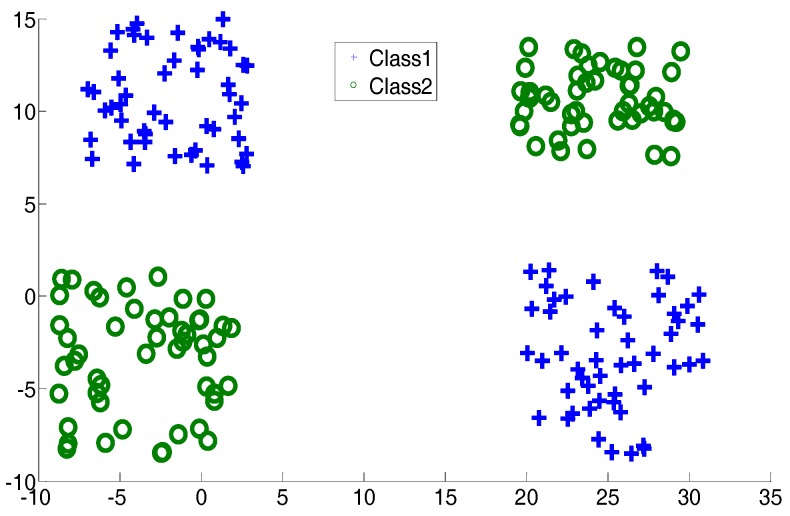
Exclusive OR dataset.

**Figure 3:**
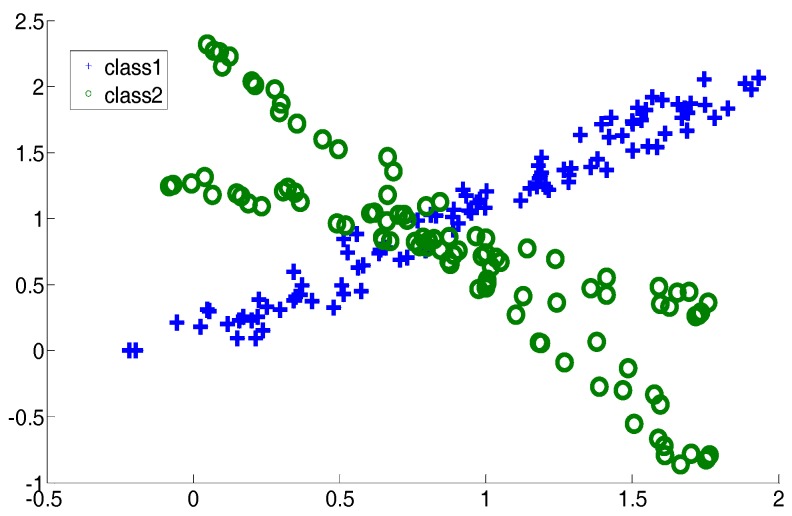
Cross plane dataset.

In this subsection, two two-dimensional synthetic datasets, an exclusive OR (XOR) dataset and a crossing line dataset, are described. They are shown in Figure 2 and Figure 3. The aim of introducing two such datasets is to simulate the complex distribution of medical data. In the experiments, for CSVH, class 1 was viewed as the patient class and class 2 was viewed as the health class. Here, besides CSVH, we conducted SVM [18,19]; local discriminative SVM (LDSVM) [25]; two SVM variants, multi-weight vector projection support vector machines (MVSVM) [31]; twin support vector machines (TWSVM) [32]; as well as a neural network, named as NN1. NN1 has three layers, including an input layer, a hidden layer and an output layer. The numbers of neurons in these three layers are two, five, and one, respectively. The activation functions of the three layers were: *f*_1_(*x*) = *x*, *f*_2_(*x*) = tansig(*x*), and *f*_3_(*x*) = *x*. An error back propagation algorithm (BP) [33], was employed to adjust the weights of NN1. For LDSVM, it consisted of an offline SVM piece and an online kNN piece. The kNN piece tuned its neighborhood size, say *k*, with the parameterization heuristic reported in [25]. Table 1 records the average training classification accuracy and testing classification accuracy over 10 dependent runs. In each run, 20% of the data were sampled randomly as training data. The accuracy is the percentage of samples that were correctly classified, and it equals the ratio of the number of samples which were correctly classified to the size of samples. In other words, *TP*, *TN*, *FP*, and *FN* denote the number of true positives, the number of true negatives, the number of false positives, and the number of false negatives, respectively; the accuracy is defined as:

$$Accuracy=\frac{TP+TN}{TP+TN+FP+FN}$$

From Table 1, we find that CSVH and TWSVM generated better results than the other classifiers. TWSVM worked well on the training data, and CSVH behaved well over the testing data. TWSVM seeks two hyperplanes that cross two classes through solving two quadratic optimization problems, which helps it to collect comprehensive structure information for the training data and produce high accuracy over the training data. CSVH learns the difference information between two classes and takes the outliers as well as overlapping-region data into consideration through constructing two customized hyperspheres. This enables CSVH to obtain the discrimination information carried by both dominated data and outliers, which benefits its generalization ability on testing data. The CSVH’s behavior verified the validation and performance of CSVH. MVSVM shares a similar spirit to TWSVM, but it generates two hyperplanes through solving two eigenvalue problems, which implies that the solution of MVSVM is not as good as that of TWSVM.

**Figure 4:**
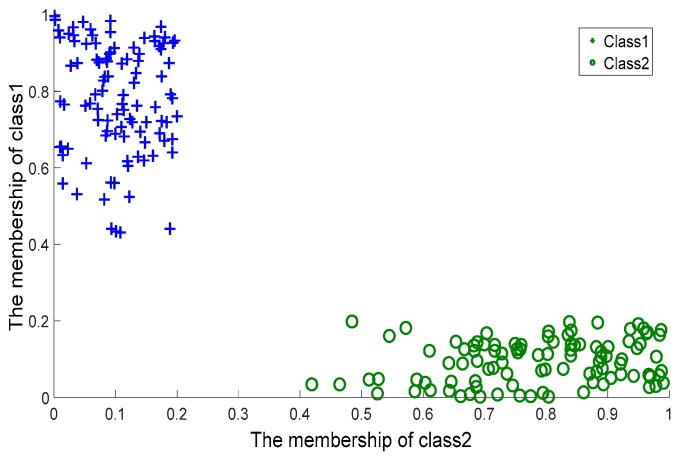
Characterized Support Vector Hyperspheres: membership values.

**Figure 5:**
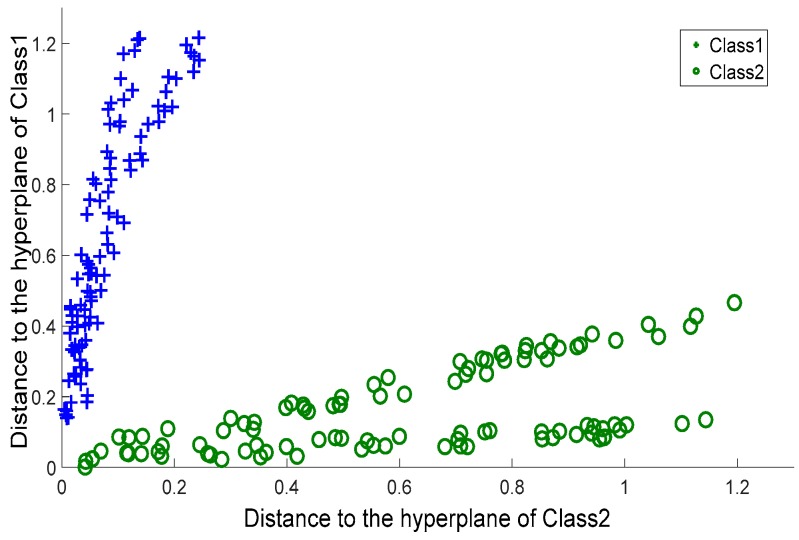
Multi-weight Vector projection Support Vector Machines: Distance values.

**Figure 6:**
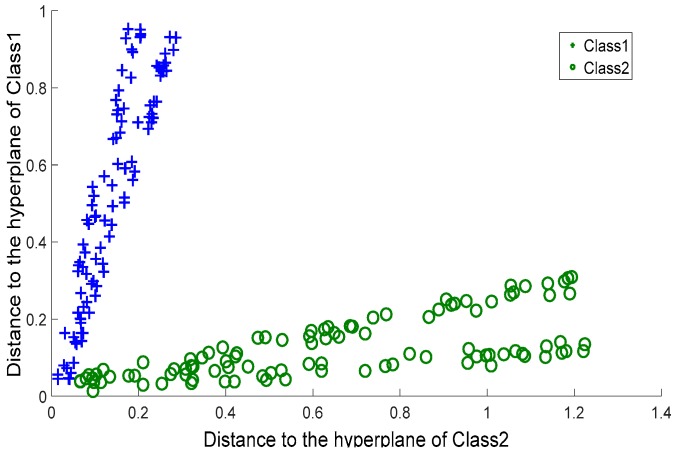
Twin Support Vector Machines: Distance values.

To further evaluate the quality of the two membership functions, *G_p_*(*Q*) and *G_h_*(*Q*), we took (*G_p_*(*Q*), *G_h_*(*Q*)) as the coordinates of *Q*, and plotted the coordinates of the data coming from the crossing line dataset on a planar system, as shown in Figure 4. For comparison, we considered MVSVM and TWSVM. MVSVM and TWSVM label a query according to the distance of the query to two hyperplanes. Therefore, for these two classifiers, we took (*d*_1_(*Q*), *d*_2_(*Q*)) as the coordinates of *Q*, where *d*_1_(*Q*) and *d*_2_(*Q*) represent the distance values of *Q* to the centers of class 1 and class 2, respectively. The corresponding planar coordinate systems of MVSVM and TWSVM are shown in Figure 5 and Figure 6.

It can be observed that the coordinates of Figure 4 are clustered more densely than those of the other two figures. This indicates that the membership values provided by CSVH are of higher indication ability and consequently, that the membership functions are of stronger discrimination ability in detecting reasonable labels than those of MVSVM and TWSVM. From Figure 5 and Figure 6, we can see that TWSVM shows higher performance than MVSVM, since the coordinates generated by TWSVM are gathered more closely than those gathered by MVSVM.

### 4.2. Experiments on Benchmark Datasets

This subsection utilizes benchmark datasets of UCI Machine Learning Repository [34] for empirical tests. Table 2 reports the average testing classification accuracy over 10 dependent runs, where in each run, 20% of the data were sampled randomly as training data, and the remaining 80% data were tested. We also list the standard deviation after the average clarification accuracy.

From Table 2, it is easy to reach a similar conclusion as set out in Section 4.1. Among the six classifiers, CSVH and TWSVM work best, and in the remaining cases, LDSVM, SVM and MVSVM follow them in turn. For the involved UCI datasets, there are common margins between the two classes, which allow SVM and LDSVM to bring all potentialities into full play to generate the qualified cutting hyperplane. Moreover, LDSVM presents higher accuracy than SVM, owing to its additional kNN component to refine the less confident decisions. In this section, MVSVM is competitive with SVM. MVSVM constructs crossing hyperplanes that could collect more discrimination information than the cutting hyperplane generated by SVM. However, MVSVM’s implementation depends on eigenvalue problems, which bring less computational cost, in exchange for decreased solution quality. In Table 2, NN1 follows the other classifiers.

The standard deviations are listed to show the stability of the classifiers. If the standard deviation is taken into consideration, CSVH has the narrower changing range than the other classifiers. This implies that the proposed CSVH is more stable than other classifiers.

We then verified whether there is statistically significance in the difference among the performance of the different classifiers. We employed a statistical test, the Friedman test [35], to conduct statistical performance analysis among the comparing classifiers. Table 3 lists the Friedman statistics *F_F_* values for each dataset at the significance level α = 0.05. For the configuration of Table 3, the corresponding critical value is 2.422. As shown in Table 3, all the Friedman statistics *F_F_* are greater than the critical value. This means that at a significance level of α = 0.05, the null hypothesis that all the comparing classifiers perform equivalently is clearly rejected, in other words, there is a statistically significant difference in accuracy among the classifiers for each dataset.

### 4.3. Experiments on Atrial Hypertrophy Datasets

For this section, two real electrocardiogram atrial hypertrophy datasets, MGH/MF [26,27] and Fantasia [27,28], acquired from medical sensors were adopted. The description of the data is presented in Section 2.

Existing solutions to atrial hypertrophy, including NNs, SVM, LDSVM, and the proposed CSVH, were evaluated. Moreover, the two SVM variants mentioned above, MVSVM and TWSVM were also included. As a binary classification tool, they fulfil the separation between the patient and health classes. To further ensure a fair comparison, we followed the NN specification in [33]. The NN described in this section is named NN2. NN2 has three layers. The numbers of neurons in the input, hidden, and output layers were 50, 10, and one, respectively. The activation functions of these three layers were the proportional function, *tansig*() function, and proportional function, respectively. In the experiments, some instances were selected randomly from two real datasets, and their corresponding vectors formed the experimental training subset. The vectors of the remaining instances formed the testing subsets.

Firstly, we observed the performance of the various approaches in the training subsets with the balanced sizes of the two classes. We denoted the size of all instances as *N*, and we selected *ρ*∙*N* instances to form the training subset, with *ρ* = 0.2, 0.4, and 0.6, respectively. In each training subset, the ratios of patient instances and healthy people instances were half and half. Table 4 reports the average testing classification accuracy as well as the standard deviation over 10 independent runs. According to Table 4, CSVH achieved higher accuracy than the other approaches. In the last section, CSVH exhibited a little advantage over TWSVM in the benchmark datasets, which verified the validation of CSVH. However, in the atrial hypertrophy datasets, CSVH exhibited an obvious improvement over TWSVM. This verified the advantage of CSVH for handling atrial hypertrophy diagnosis over the peer approaches. CSVH was followed by LDSVM, TWSVM, SVM, MVSVM, and NN2 in turn. The outperformance of CSVH is attributed to the formulation of the two characterized hyperspheres. CSVH takes the characters of the two classes into consideration to strengthen the diversity of patient instances through generating a weighted hypersphere, and to pursue the maximum separation between healthy people instances and patient instances through keeping the furthest distance between two hyperspheres. This enables the resulting membership functions to be encoded with the discrimination information that is customized to the two classes. In addition, rejection cases are handled by CSVH through computing the angle between the query and outliers, as well as the overlapping-region data. Therefore, CSVH tends to produce reasonable decisions. LDSVM presented lower accuracy than CSVH, because of its inherent difficulty in addressing overlapping-region data. Although it is equipped with the refined component kNN to modify the less confident decisions of the SVM, it should be kept in mind that the metric on which kNN works is derived from the SVM hyperplane function. In the datasets with overlapping classes, the SVM hyperplane function would be distorted by the bias introduced by the overlapping data. Consequently, the quality of the resulting metric would be affected, which would then affect the behavior of the kNN as well. As discussed above, TWSVM provided better performance than SVM. According to empirical evidence, SVM and MVSVM are competitive in common benchmark datasets, but the former behaves better than the latter in more cases. This is because SVM solves the quadratic problems while MVSVM solves two eigenvalue problems. In atrial hypertrophy data, a complex data environment, the quality optimization problem makes classification accuracy important. The advantage of solutions to quadratic problems helps SVM present higher accuracy than MVSVM in most cases. As for NN2, its analysis was similar to the above section.

In order to show a statistically significant difference, a Wilcoxon signed-ranks test [35] was employed. The Wilcoxon signed-ranks test is a statistical test that ranks the difference in performance of two classifiers for each dataset. We summarized the statistical test results at the significance level α = 0.05 and report them in Table 5. Generally, if the *p*-value of the test is less than 0.05, it indicates there are statistically significant differences in accuracy between the two classifiers. It can be observed from Table 5 that the p-values are less than 0.05, which indicates that the proposed CSVH outperforms the competitor in statistical significance.

**Figure 7:**
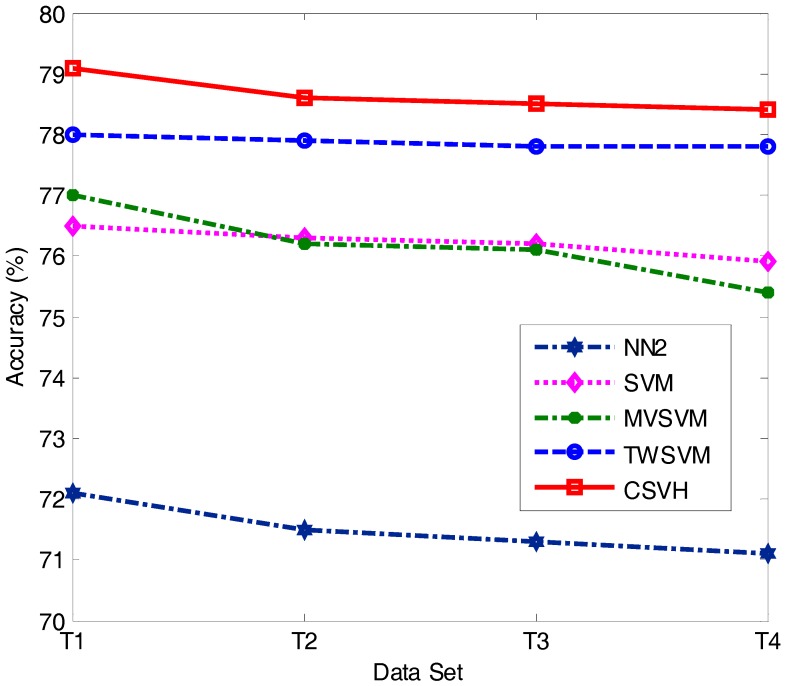
Classification accuracy of T1, T2, T3, and T4.

**Figure 8:**
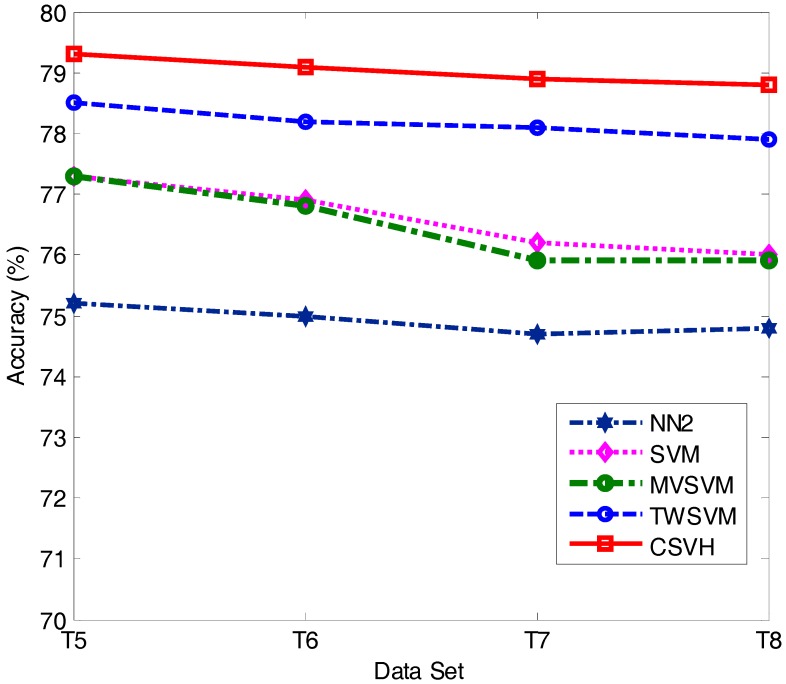
Classification accuracy of T5, T6, T7, and T8.

It is typical that in clinical practice, between the patient and health classes, the size of one side is smaller than the other. We thus considered the cases where the sizes of two classes were imbalanced. We simulated such scenarios with the training subsets specified in Table 6, where ‘#’ represents the number of instances of the corresponding class; T1, T2, … and T8 represent eight training cases. LDSVM is equipped with kNN, and is sensible to the size of training data. Thus, except LDSVM, we considered the diagnosis accuracy of the other four classifiers as the training data size changed. The diagnosis accuracy of the tested approaches is shown in Figure 7 and Figure 8.

From the experimental results, we found that the accuracy values of the five approaches reported in Figure 7 are lower than those in Figure 8. This suggests that decreasing the patient class size has more influence on the diagnosis results than decreasing the health class size. This is because the instances of the patient class are of higher diversity, which means a small amount of patient instances would fail to provide comprehensive class structure information. Consequently, the resulting classifier is unable to hold sufficient discriminative knowledge to distinguish patient class vectors from health class vectors. On the contrary, in the experiments involved in our paper, the distribution of the health instances was relatively closely gathered. This allows a small number of health instances to reveal sufficient discriminative knowledge concerned with the health class.

Another observation is that in both Figure 7 and Figure 8, CSVH presents consistently higher accuracy than its four peers. This further verifies the validation and higher performance of CSVH in atrial hypertrophy diagnosis. Moreover, by comparing Figure 7 and Figure 8 with Table 4, we observe that CSVH and TWSVM are less affected by imbalanced training data than the other three approaches. The reason for this is that CSVH and TWSVM construct two characterized hyperspheres and two crossing hyperplanes, respectively, for the two classes. Thanks to the customization to the individual class, these models can flexibly adapt to the change in class size. MVSVM also generates two crossing hyperplanes for the two classes; however, its model formulation is implemented by solving eigenvalue problems. Compared with CSVH and TWSVM, which solve quadratic optimization problems, MVSVM’s solutions are not as stable as those of CSVH and TWSVM. As for SVM, it constructs a hyperplane with the margin between two classes. An imbalance of the two sides would attract the hyperplane away from the central sites of the margin, which would reduce the width of the separation of the hyperplane and affect the classification accuracy.

The standard deviations are listed in Table 7, and it can be observed that the CSVH is more stable than the other classifiers. It can be observed from Table 7 that CSVH is more stable than other SVM-based methods.

Finally, we will provide a brief discussion of time complexity. We denoted the size of a dataset as *N*, and supposed the size of each class as *N*/2. CSVH’s time consumption was then 2∙*O*((*N*/2)^3^), which is consumed in executing two quadratic optimization problems. TWSVM and MVSVM were of the same time complexity as CSVH. SVM consumed high cost with *O*(*N*^3^), and NN2’s time consumption was unexpected because it depends on many factors, including the structure of the network (number of layers and neurons) and the termination condition (using pre-specified iteration times or an error threshold), among others. Taking both the performance and cost into consideration, CSVH is more desirable than its competitors.

## 5. Conclusions and Ongoing Work

Computer-aided diagnosis for atrial hypertrophy using sensor data monitored from medical sensors is a challenging problem owing to the absence or poor quality of training data and the confused difference between the healthy subjects and patients. For this reason, this paper proposed a new atrial hypertrophy diagnosis method based on characterized support vector hyperspheres (CSVH), which generates two hyperspheres for the two classes. The patient class hypersphere is characterized by a weighted model formulation, with the aim of taking the diversity of patient instances into consideration. The health class hypersphere is characterized by the furthest distance to the patient class, with the aim of achieving the maximum separation from the patient class. Based on the two hyperspheres, membership functions are defined. If a query is rejected by the two classes, it is labelled according to the information of the outliers and the overlapping-region data. The balance coefficient is parameterized adaptively. The experimental results demonstrate the higher performance of CSVH over its peers on real atrial hypertrophy datasets and its competitive behavior in common classification tasks in comparison to state-of-the-art methods. Techniques for combining Bayesian networks [36] with CSVH are under development to integrate the prior knowledge of medical experts, and work to develop some techniques such as ensemble or information fusion to further improve accuracy is also ongoing. We will also study the distribution of atrial hypertrophy datasets and study how to show or visualize the high dimensional distribution of the atrial data. Refining CSVH details and extending CSVH to solving multi-classification problems is also work that will be concentrated on in the future.

## Figures and Tables

**Table 1:** Classification accuracy of the two datasets (%).

Classifier	XOR Dataset		Crossing Line	
	Training	Testing	Training	Testing
NN1	95.2	93.5	97.7	97.6
SVM	95.7	93.6	96.9	96.2
LDSVM	96.1	94.2	97.1	96.5
MVSVM	96.3	93.9	97.4	96.0
TWSVM	**97.3**	**96.3**	**98.3**	97.0
CSVH	96.9	**96.3**	98.1	**97.4**

**Table 2:** Comparison of accuracy (%).

Dataset	NN1	SVM	MVSVM	TWSVM	LDSVM	CSVH
Blood transfusion	81.5 ± 3.9	82.0 ± 3.1	83.1 ± 4.2	84.0 ± 2.6	83.7 ± 3.7	**84.9** ± 4.1
Ionosphere	92.6 ± 3.1	94.8 ± 4.7	94.1 ± 5.1	**95.7** ± 4.3	95.0 ± 4.1	95.3 ± 3.0
Breast Cancer	95.3 ± 2.8	96.0 ± 3.6	95.7 ± 4.3	96.5 ± 3.4	96.6 ± 3.8	**97.4** ± 2.9
SPECTF heart	93.1 ± 3.6	93.2 ± 4.1	93.7 ± 3.1	97.2 ± 3.0	95.1 ± 4.4	**97.3** ± 3.4
Liver	72.6 ± 3.8	74.6 ± 4.2	74.1 ± 4.2	75.0 ± 2.9	74.4 ± 2.8	**75.7 ±** 2.7
Australian	85.2 ± 4.1	86.2 ± 3.3	87.0 ± 3.6	**87.9** ± 3.5	87.3 ± 4.3	87.7 ± 3.5
Diabetes	75.1 ± 3.5	75.3 ± 3.9	76.2 ± 2.6	**78.4** ± 4.2	77.2 ± 3.6	78.1 ± 3.1

**Table 3:** *F_F_* values of the Friedman test among the accuracies for each dataset (significance level α = 0.05).

Dataset	*F_F_*
Blood transfusion	44.8571
Ionosphere	36.1714
Breast Cancer	39.9429
SPECTF heart	45.8857
Liver	41.0286
Australian	32.7429
Diabetes	41.2571

**Table 4:** Diagnosis accuracy on atrial hypertrophy data (%).

Training Subset Ratio	NN	SVM	MVSVM	TWSVM	LDSVM	CSVH
*ρ* = 0.2	75.0 ± 2.7	77.5 ± 3.7	77.7 ± 3.5	78.1 ± 3.0	78.7 ± 3.6	**79.7** ± 3.3
*ρ* = 0.4	75.2 ± 3.2	77.8 ± 4.0	77.2 ± 2.8	78.3 ± 4.2	78.5 ± 3.0	**79.3** ± 2.7
*ρ* = 0.6	74.1 ± 3.9	76.2 ± 4.3	76.0 ± 4.3	77.9 ± 3.5	78.0 ± 4.1	**78.9** ± 3.1

**Table 5:** *p*-Values of the Wilcoxon signed-ranks test between CSVH and other classifiers (significance level α = 0.05).

Training Subset Ratio	CSVH vs. NN	CSVH vs. SVM	CSVH vs. MVSVM	CSVH vs. TWSVM	CSVH vs. LDSVM
*ρ* = 0.2	0.002	0.002	0.0039	0.0059	0.0156
*ρ* = 0.4	0.0039	0.0098	0.0059	0.0254	0.0273
*ρ* = 0.6	0.002	0.0237	0.0098	0.0195	0.0488

**Table 6:** Imbalanced training dataset details.

	# Patient	# Health
T1	5	20
T2	5	25
T3	5	30
T4	5	35
T5	30	5
T6	45	5
T7	60	5
T8	75	5

**Table 7:** The standard deviations of the classifiers on T1-T8.

Dataset	NN1	SVM	MVSVM	TWSVM	CSVH
T1	3.1	4.2	3.9	4.0	3.2
T2	2.8	4.1	3.2	3.5	3.0
T3	3.7	3.8	3.7	4.5	2.7
T4	3.3	4.0	4.3	4.1	3.6
T5	3.6	4.2	2.9	4.4	3.7
T6	2.6	4.0	3.4	3.6	3.2
T7	3.7	3.9	3.3	4.0	2.8
T8	3.8	4.6	3.7	3.8	3.6
